# Tyrosinase inhibitory activity of flavonoids from *Artocarpus heterophyllous*

**DOI:** 10.1186/s13065-016-0150-7

**Published:** 2016-01-29

**Authors:** Hai Xuan Nguyen, Nhan Trung Nguyen, Mai Ha Khoa Nguyen, Tho Huu Le, Truong Nhat Van Do, Tran Manh Hung, Mai Thanh Thi Nguyen

**Affiliations:** Faculty of Chemistry, University of Science, 227 Nguyen Van Cu, District 5, Ho Chi Minh, Vietnam; Cancer Research Laboratory, Vietnam National University, 227 Nguyen Van Cu, District 5, Ho Chi Minh, Vietnam; Department of Biomedical Sciences, Institute for Research and Executive Education (VNUK), The University of Danang, 41 Le Duan, Haichau District, Danang, Vietnam

**Keywords:** *Artocarpus heterophyllous*, Flavonoids, Chalcones, Tyrosinase inhibitors

## Abstract

**Background:**

Tyrosinase is an oxidoreductase that is very important in medicine and cosmetics because the excessive production of melanin causes hyperpigmentation. The development of novel, effective tyrosinase inhibitors has long been pursued. In preliminary tests, we found that an extract of the wood of *Artocarpus heterophyllous* (AH) potently inhibited tyrosinase activity.

**Results:**

Two new flavonoids, artocaepin E (**1**) and artocaepin F (**2**), were isolated from the wood of AH, together with norartocarpetin (**3**), artocarpanone (**4**), liquiritigenin (**5**), steppogenin (**6**), and dihydromorin (**7**). Their structures were elucidated using one-dimensional (1D) and two-dimensional (2D) nuclear magnetic resonance (NMR) and mass spectrometry. The absolute configuration of **2** was determined from the circular dichroism (CD) spectrum. Artocarpanone (**4**) had the most potent tyrosinase inhibitory effect, with an IC_50_ of 2.0 ± 0.1 μM, followed by artocaepin E (**1**) and steppogenin (**6**), with IC_50_ values of 6.7 ± 0.8 and 7.5 ± 0.5 μM, respectively. A kinetic investigation indicated that **1** showed competitive inhibition, with an inhibition constant (*K*_i_) of 6.23 μM.

**Conclusions:**

These results demonstrate that extracts of the wood of AH and its phytochemical constituents are potential sources for skin-whitening agents.

**Graphical abstract:**
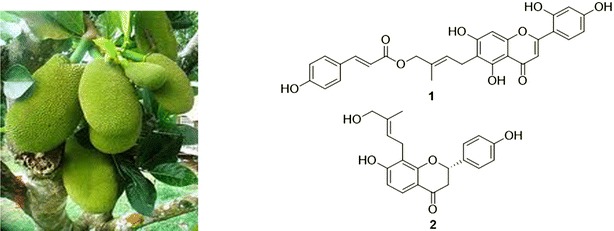
Artocarmin E (1) and artocarmin F (2) were isolated from the wood of *Artocarpus heterophyllous*. Their structures were elucidated using nuclear magnetic resonance analysis and mass spectrometric methods

**Electronic supplementary material:**

The online version of this article (doi:10.1186/s13065-016-0150-7) contains supplementary material, which is available to authorized users.

## Background

Tyrosinase is a key enzyme in mammalian melanin synthesis. It catalyzes the first step in two reactions of melanin synthesis: the hydroxylation of l-tyrosine to 3,4–dihydroxyphenylalanine (l-DOPA) and the oxidation of l-DOPA to dopaquinone. This *o*-quinone is a highly reactive compound that can polymerize spontaneously to form melanin [1]. In mammals, melanin protects the skin from ultraviolet (UV) damage by absorbing UV sunlight and removing reactive oxygen species. However, the production of abnormal melanin pigmentation is an esthetic problem in humans [2]. Therefore, the development of high-performance tyrosinase inhibitors is much needed.

*Artocarpus heterophyllous* Lam belongs to the family Moraceae and is popularly known as jackfruit; it is distributed widely in tropical and subtropical regions of Asia. In Vietnam, this plant is known as “Mit,” and is cultivated for its edible fruits, while the wood has been used for its anti-inflammatory, antioxidant, and antiaging effects [3]. In preliminary tests, we found that an extract of the wood of AH strongly inhibited tyrosinase activity. This plant is a rich source of prenylated flavonoids and their derivatives. Numerous compounds have been isolated from AH, including prenylated flavonoids, chalcones, and simple polyphenols. These possess various biological activities, including cytotoxic, tyrosinase inhibitory, anti-inflammatory, and antioxidant effects [4–6]. The present phytochemical investigation of the wood of AH led to the isolation of two new flavonoids (**1**, **2**) and five known compounds (**3**−**7**). In this paper, we report the isolation and structure elucidation of these isolated compounds, as well as their tyrosinase inhibitory activities.

## Results and discussion

### Chemistry

**Fig. 1:**
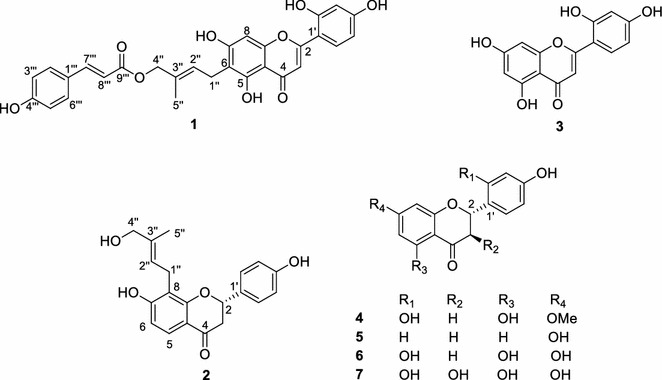
Isolated compounds from *A. heterophyllous* (**1** − **7**)

Dried *A. heterophyllous* wood was extracted in methanol, and the obtained extract was successively partitioned into *n*-hexane, chloroform (CHCl_3_), ethyl acetate (EtOAc), butanol (*n*-BuOH), and water. Repeated silica gel and reverse-phase column chromatography of the CHCl_3_ fraction afforded two new flavonoids (**1**−**2**) and five known ones (**3**−**7**). The chemical structures of the known compounds were determined based on ^1^H- and ^13^C-NMR analyses and confirmed by comparison with reported spectra as norartocarpetin (**3**) [7], artocarpanone (**4**) [8], liquiritigenin (**5**) [9], steppogenin (**6**) [7], and dihydromorin (**7**) [10] (Fig. 1).

**Fig. 2:**
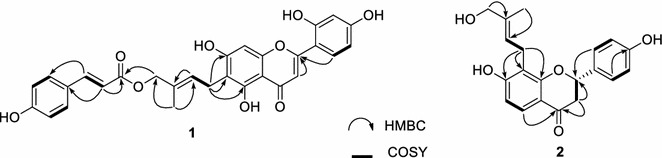
Selected key HMBC and ^1^H-^1^H COSY correlations for **1** and **2**

Compound **1** was obtained as a yellow amorphous solid. High-resolution electrospray ionization mass spectrometry (HR-ESI–MS) gave a pseudo-molecular peak at *m/z* 517.1487 [M + H]^+^ (calcd. for 517.1499), consistent with the molecular formula C_29_H_24_O_9_. The infrared (IR) spectrum suggested the presence of hydroxy (3410 cm^−1^), phenyl (1600, 1455 cm^−1^), and carbonyl (1710, 1700 cm^−1^) groups. The ^1^H NMR spectrum of **1** included signals due to two sets of *ortho*-coupled aromatic protons at *δ*_H_ 7.53 (2H, d, *J* = 8.7 Hz, H-2′′′ and 6′′′) and 6.78 (2H, d, *J* = 8.7 Hz, H-3′′′ and 5′′′), three aromatic protons of the typical *ABX* system at *δ*_H_ 6.49 (1H, d, *J* = 2.4 Hz, H-3′), 7.73 (1H, d, *J* = 8.8 Hz, H-6′), and 6.44 (1H, dd, *J* = 8.8, 2.4 Hz, H-5′), an isolated aromatic proton at *δ*_H_ 6.51 (1H, s, H-8), two *trans* olefinic protons at *δ*_H_ 7.54 (1H, d, *J* = 16.0 Hz, H-7′′′) and 6.39 (1H, d, *J* = 16.0 Hz, H-8′′′), and two isolated olefinic protons at *δ*_H_ 6.98 (1H, s, H-3) and 5.55 (1H, t, *J* = 7.2 Hz, H-2′′), together with one methyl, one methylene, one oxymethylene, and a characteristic signal of a hydrogen-bonded hydroxyl proton at *δ*_H_ 13.34 (1H, s, 5-OH) (Table 1). In comparison, the [13] C NMR and distortionless enhancement by polarization transfer (DEPT) spectrum of **1** contained 29 carbon signals, including a ketone carbonyl, an ester carbonyl, six olefinic, a methyl, a methylene, an oxymethylene, and 18 aromatic carbons (Additional file 1). The data were similar to that of artocaepin D, which was isolated from the same plant [11], except for the presence of signals of a set of resonances due to a *trans*-*p*-coumaroyl unit, which was confirmed by the ^1^H–^1^H correlation spectroscopy (COSY) and heteronuclear multiple bond correlation (HMBC) spectra (Fig. 2). This moiety was located at C-4″ by the HMBC correlation of H-4″ (*δ*_H_ 4.51, 2H, s) with the C-9′″ (*δ*_C_ 166.5) ester carbonyl carbon of the *trans*-*p*-coumaroyl group. The nuclear Overhauser effect spectroscopy (NOESY) correlations of H-1″ with H-5″ and of H-2″ with H-4″ indicated an *E*-configuration for the C-2″/C-3″ double bond of **1**. Therefore, the structure of artocaepin E was concluded to be **1**.

**Table 1:** ^1^H (500 MHz, *J* in Hz) and ^13^C (125 MHz) NMR data for **1** and **2**

Position	**1** ^a^		**2** ^b^	
	*δ*_H_	*δ*_C_	*δ*_H_	*δ*_C_
2		161.7	5.44 dd (12.8; 3.0)	80.4
3	6.98 s	106.8	2.70 dd (16.7; 3.0)3.00 dd (16.7; 12.8)	44.6
4		182.0		191.0
5		158.4	7.59 d (8.6)	126.4
6		109.7	6.63 d (8.6)	110.5
7		161.7		162.4^c^
8	6.51 s	93.2		115.5
9		155.3		162.1^c^
10		103.3		130.9
1′		108.7		131.6
2′		158.8	7.42 d (8.6)	128.8
3′	6.49 d (2.4)	103.3	6.90 d (8.6)	116.2
4′		161.7		158.6
5′	6.44 dd (8.8; 2.4)	108.1	6.90 d (8.6)	116.2
6′	7.73 d (8.8)	129.8	7.42 d (8.6)	128.8
1″	3.30 d (7.2)	20.8	3.38 d (7.3)	22.4
2″	5.55 t (7.2)	126.4	5.49 t (7.3)	122.8
3″		130.2		136.3
4″	4.51 s	69.0	3.87 s	68.5
5″	1.79 s	13.9	1.65 s	13.9
1'''		125.1		
2''', 6'''	7.53 d (8.7)	130.4		
3''', 5'''	6.78 d (8.7)	115.8		
4'''		159.9		
7'''	7.54 d (16.0)	144.9		
8'''	6.39 d (16.0)	114.2		
9'''		166.5		
5-OH	13.34			

^a^In DMSO-*d*_6_

^b^In acetone-*d*_6_

^c^These signals may interchange

Artocaepin F (**2**) was isolated as a yellow amorphous solid. The molecular formula of **2** was determined to be C_20_H_20_O_5_ by HR-ESI–MS. The IR spectrum showed absorption bands of hydroxy (3400 cm^−1^), phenyl (1600, 1450 cm^−1^), and ketone (1705 cm^−1^) groups. The ^1^H NMR spectra of **2** (Table 1) showed signals for two sets of *ortho*-coupled aromatic protons at *δ*_H_ 7.42 (2H, d, *J* = 8.6 Hz, H-2′, and H-6′) and 6.90 (2H, d, *J* = 8.6 Hz, H-3′ and H-5′), and others at *δ* 7.59 (1H, d, *J* = 8.6 Hz, H-5) and 6.63 (1H, d, *J* = 8.6 Hz, H-6), together with two methylene signals at *δ*_H_ 2.70 (1H, dd, *J* = 16.7, 3.0 Hz, H-3a) and 3.00 (1H, dd, *J* = 16.7, 12.8 Hz, H-3b), and one oxymethine at *δ*_H_ 5.44 (1H, dd, *J* = 12.8, 3.0 Hz, H-2), which are typical of the flavanone skeleton [9]. The ^13^C NMR and DEPT spectrum of **2** displayed 20 carbon signals, including 15 carbon signals due to the flavanone skeleton and five belonging to a 4-hydroxyprenyl moiety (Fig. 1). The 4-hydroxyprenyl group was located at C-8 based on the HMBC correlations between H-1″ (*δ*_H_ 3.38, 2H, d, *J* = 7.3 Hz) and C-7, C-8, C-8a, and of H-2″ (*δ*_H_ 5.49, 1H, t, *J* = 7.3 Hz) with C-8 (Fig. 2). The NOESY correlations of H-1″ with H-5″ and of H-2″ with H-4″ indicated that the configuration of the C-2″/C-3″ double bond of **2** was the same as that of **1**. Finally, the absolute configuration at C-2 was considered to be *S* according to the results of the CD spectroscopic analysis, which showed negative and positive Cotton effects at 290 and 334 nm, respectively [12]. From this spectroscopic evidence, the structure of artocaepin F was concluded to be **2**.

### Biological assay

The tyrosinase inhibitory activity of all isolated compounds (**1–7**) was tested [11]. Kojic acid, a well-known tyrosinase inhibitor currently used as a cosmetic skin-whitening agent, was used as a positive control. Of the tested compounds, artocarpanone (**4**) had the most potent inhibitory effect against tyrosinase, with an IC_50_ of 2.0 ± 0.1 μM, followed by artocaepin E (**1**) and steppogenin (**6**), with IC_50_ values of 6.7 ± 0.1 and 7.5 ± 0.5 μM, respectively (Table 2). Liquiritigenin (**5**) also showed significant concentration-dependent inhibition, with an IC_50_ of 22.0 ± 2.5 μM; this compound showed moderate inhibitory activity compared to the above compounds. However, it showed more potent inhibitory activity than kojic acid, which inhibited tyrosinase with an IC_50_ of 44.6 ± 0.4 μM (Table 2). The other compounds, artocaepin F (**2**), norartocarpetin (**3**), and dihydromorin (**7**), showed very weak inhibitory activity, with IC_50_ values over 50 μM.

**Table 2:** Tyrosinase inhibitory activity of the isolated compounds **1**−**7**

Compounds	IC_50_ (µM)^a^
**1**	6.7 ± 0.8
**2**	>50
**3**	>50
**4**	2.0 ± 0.1
**5**	22.0 ± 2.5
**6**	7.5 ± 0.5
**7**	>50
Kojic acid^b^	44.6 ± 0.4

^a^ The assay was executed in triplicate

^b^ Positive control used for enzymatic inhibition assay

**Fig. 3:**
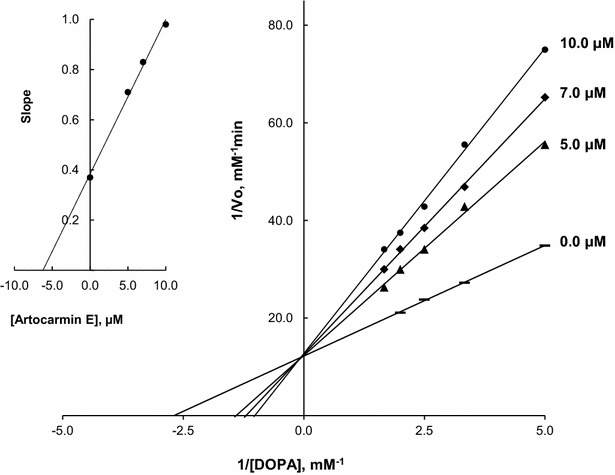
Lineweaver–Burk plots for type of inhibition of mushroom tyrosinase (10 U/mL) by artocaepin E (**1**) for the catalysis of l-DOPA (0.2, 0.3, 0.4, 0.5, and 0.6 mM) at 30 °C, pH 6.8. Concentration of these compounds for curves I_0.0_, I_5.0_, I_7.0_, and I_10.0_ were 0.0, 5.0, 7.0, and 10.0 μM, respectively. The* inset* represents the plot of these compounds for determining the inhibition constant (*K*i). The line is drawn using a linear lest squares fit

Further study examined the inhibitory mechanism of artocaepin E (**1**), which strongly inhibited tyrosinase activity. To determine the type of enzyme inhibition and the inhibition constant for an enzyme-inhibitor complex, the mechanism was analyzed by Lineweaver–Burk plots. The results indicated that **1** displayed competitive inhibition, with an inhibition constant (*K*_i_) of 6.23 μM (Fig. 3).

On close inspection of the inhibitory activity exerted by these compounds, the following biological profile of the structure–activity relationship was deduced. In terms of the flavone skeleton, compounds **1** and **3** are derivative of apigenin, a common flavone in plants; however, the presence of one hydroxyl group at C-2′, a *trans*-*p*-coumaroyl unit connected to the hydroxyprenyl through an ester linkage at C-6 of the apigenin skeleton in **1**, led to significantly stronger inhibitory activity than that of **3** (6.7 vs. >50 μM). This suggests that the absence of the side-chain at C-6 of the B-ring leads to a significant loss of activity, and the presence of a side-chain such as *trans*-*p*-coumaroyl connected to the hydroxyprenyl may positively influence the tyrosinase inhibitory activity. Regarding the flavanone skeleton, artocarpanone (**4**), which possesses a methoxyl group at C-7 of ring A, had the strongest inhibitory activity. Steppogenin (**6**) shares the same structure as **4**, except for the hydroxyl group at C-7; however, it had 3.75-fold higher inhibitory activity than **4**. In comparison, dihydromorin (**7**), which has four hydroxyl groups at C-2′, C-3, C-5, and C-7, had weak activity. These results imply that the methoxyl and hydroxyl groups in the main flavanone skeleton play an important role in tyrosinase inhibition.

## Methods

### General procedure

Optical rotations were recorded on a JASCO DIP-140 digital polarimeter. CD measurements were carried out on a JASCO J-805 spectropolarimeter. IR spectra were measured with a Shimadzu IR-408 spectrophotometer in CHCl_3_ solution. NMR spectra were taken on a Bruker Advance III 500 spectrometer (Brucker Biospin) with tetramethylsilane (TMS) as an internal standard, and chemical shifts are expressed in *δ* values. HR-ESI–MS measurements were carried out on a Bruker microTOF-QII spectrometer. Column chromatography was performed with BW-820MH Si gel (Fuji Silisia, Aichi, Japan). Analytical and preparative TLC was carried out on precoated Merk Kiesegel 60F_254_ or RP-18F_254_ plates (0.25 or 0.5 mm thickness).

### Chemicals

Tyrosinase (EC 1.14.18.1) from mushroom (3933 U/mL) and l-dihydroxyphenylalanine (l-DOPA) were obtained from Sigma Chemical Co. (St. Louis, MO, USA). Kojic acid and DMSO were purchased from Merck (Darmstadt, Germany). Other chemicals were of the highest grade available.

### Plant material

The wood of *A. heterophyllous* was collected at the Seven-Mountain area, An Giang province, Vietnam in August 2010. The plant was identified by Ms. Hoang Viet, Faculty of Biology, University of Science, Vietnam National University-Hochiminh City. The voucher sample of the wood part (AN-2985) is preserved at Department of Analytical Chemistry, Faculty of Chemistry, University of Science, Vietnam National University-Hochiminh City.

### Extraction and isolation

The dried powder of wood of *A. heterophyllous* (5.8 kg) was extracted with MeOH (15 L, reflux, 3 h, × 3) to yield a MeOH extract. The extract was partitioned between EtOAc and water to give an EtOAc-soluble fraction (64.2 g). The EtOAc-soluble fraction was subjected to silica gel column chromatography with acetone−hexane to give six fractions fr. 1–6. Fraction 6 was chromatographed further using a MeOH−CHCl_3_ gradient system to afford four subfractions fr. 6.1–6.4. Sub-fraction 6.2 was chromatographed further using MeOH−CHCl_3_ gradient system, with final purification effected by preparative TLC with 2 % MeOH−CHCl_3_, to give **4** (6.5 mg) and **5** (20.8 mg). Subfraction 6.3 was separated by preparative TLC with 5 % MeOH−CHCl_3_ to give **1** (5.0 mg), and **2** (5.3 mg), and **3** (8.5 mg). Subfraction 6.4 was re-chromatographed on silica gel with 7 % MeOH−CHCl_3_, followed by final purification using preparative TLC with 40 % acetone−hexane, to give **6** (8.0 mg), and **7** (7.5 mg).

Artocaepin E **(1)**: pale yellow, amorphous solid; IR *ν*_max_ (CHCl_3_) 3395, 1655, 1615, 1400 cm^−1^; ^1^H and ^13^C NMR (DMSO-*d*_6_ 500 MHz) see Table 1; HR-ESI–MS *m/z* 517.1487 (calcd. for C_29_H_25_O_9_ [M + H]^+^, 517.1499).

Artocaepin F **(2)**: yellowish gum; [α]_D_^25^ −10.0° (*c* 1.0, C_2_H_5_OH); IR *ν*_max_ (CHCl_3_) 3365, 1630, 1600, 1510 cm^−1^; ^1^H and ^13^C NMR (acetone-*d*_6_ 500 MHz) see Table 1; HR-ESI–MS *m/z* 363.1224 (calcd. for C_20_H_20_O_5_Na [M + Na]^+^, 363.1208).

## Tyrosinase inhibitory assay

All the samples were first dissolved in DMSO and used for the actual experiment at concentrations of 100^-1^ µg/mL (or µM for pure compounds). The tyrosinase inhibitory activity assay was performed as previously described by Arung et al. [13]. The assay mixtures consisting of 1900 µL of test solution in 0.1 M phosphate buffer pH 6.8 and 100 µL of enzyme solution (15 U/mL in 0.1 M phosphate buffer pH 6.8) was prepared immediately before use. After preincubation at room temperature for 30 min, the reaction was initiated by the addition of 1000 µL of substrate solution (1.5 mM l-DOPA in 0.1 M phosphate buffer pH 6.8). The assay mixture was incubated at room temperature for 7 min, and the absorbance at 475 nm was measured with a Shimadzu UV-1800 spectrophotometer. Kojic acid, a known tyrosinase inhibitor, was used as positive control. Tyrosinase inhibitory activity was expressed as the percentage inhibitory of enzyme tyrosinase in the above assay system, calculated as (1 − *B*/*A*) × 100, where *A* and *B* are the activities of the enzyme without and with test material. IC50 values were calculated from the mean values of data from four determinations.

## Inhibition mechanism

The procedure for determination of the inhibition mechanism was similar to that for determination of IC_50_, except that uninhibited and inhibited reactions were observed for three different concentrations of l-DOPA (0.2, 0.3, 0.4, 0.5, and 0.6 mM) at 30 °C in 0.1 M phosphate buffer pH 6.8. The dependence of absorbance (475 nm) on time was measured, and the reaction rate was calculated for all reactions (uninhibited and inhibited). Then, a Lineweaver–Burk plot was constructed, and *K*_m_ and *V*_m_ values were calculated. Each measurement was performed in duplicate.

## Conclusions

In this study, we identified two new flavonoids from the wood of AH, artocaepin E (**1**) and artocaepin F (**2**), together with five known compounds: norartocarpetin (**3**), artocarpanone (**4**), liquiritigenin (**5**), steppogenin (**6**), and dihydromorin (**7**). Regarding tyrosinase inhibition, artocarpanone (**4**) had the greatest inhibitory effect, followed by artocaepin E (**1**) and steppogenin (**6**). Liquiritigenin (**5**) also showed significant concentration-dependent inhibition. Kinetic studies indicated that the new active compound artocaepin E (**1**) displayed competitive inhibition. These results suggest that these compounds may serve as structural templates for the design and development of novel tyrosinase inhibitors as effective anti-browning agents in cosmetics.

## Additional file

10.1186/s13065-016-0150-7 One-dimensional (1D) and two-dimensional (2D) nuclear magnetic resonance (NMR) andmass spectrometry (MS) of compounds 1-2
