# Supplementary material for: Tyrosinase inhibitory activity of flavonoids from Artocarpus heterophyllous
Source: Chem Cent J. 2016 Jan 29;10:2. doi: 10.1186/s13065-016-0150-7 (PMC4734850; doi:10.1186/s13065-016-0150-7)

Tyrosinase Inhibitory Activity of Flavonoids from *Artocarpus heterophyllus*

Hai Xuan Nguyen^1^, Nhan Trung Nguyen^1,2^, Mai Ha Khoa Nguyen^1^, Tho Huu Le^1^, Truong Nhat Van Do^1^, Tran Manh Hung^3*^, and Mai Thanh Thi Nguyen ^1,2^*

**Affiliation**

^1^ Faculty of Chemistry, University of Science, Vietnam National University-Hochiminh City, Vietnam

^2^ Cancer Research Laboratory, Vietnam National University-Hochiminh City, Vietnam

^3^ Department of Biomedical Sciences, Institute for Research & Executive Education (VNUK), The University of Danang, 41 Le Duan, Haichau District, Danang city, Vietnam

**^*^Correspondences:** [hung.tran@vnuk.edu.vn](mailto:hung.tran@vnuk.edu.vn) (TMH); [nttmai@hcmus.edu.vn](mailto:nttmai@hcmus.edu.vn) (MTTN)

**List of Figures**

**Fig. 1S** ^1^H NMR spectrum of compound **1** (DMSO-*d_6_*, 500 MHz)

**Fig. 2S** ^13^C NMR spectrum of compound **1** (DMSO-*d_6_*, 125 MHz)

**Fig. 3S** DEPT spectrum of compound **1** (DMSO-*d_6_*)

**Fig. 4S** COSY spectrum of compound **1** (DMSO-*d_6_*)

**Fig. 5S** HSQC spectrum of compound **1** (DMSO-*d_6_*)

**Fig. 6S** HMBC spectrum of compound **1** (DMSO-*d_6_*)

**Fig. 7S** NOESY spectrum of compound **1** (DMSO-*d_6_*)

**Fig.8S** MS spectrum of compound **1**

**Fig. 9S** ^1^H NMR spectrum of compound **2** (CD_3_COCD_3_, 500 MHz)

**Fig. 10S** ^13^C NMR spectrum of compound **2** (CD_3_COCD_3_, 125 MHz)

**Fig. 11S** DEPT spectrum of compound **2** (CD_3_COCD_3_)

**Fig. 12S** COSY spectrum of compound **2** (CD_3_COCD_3_)

**Fig. 13S** HSQC spectrum of compound **2** (CD_3_COCD_3_)

**Fig. 14S** HMBC spectrum of compound **2** (CD_3_COCD_3_)

**Fig. 15S** NOESY spectrum of compound **2** (CD_3_COCD_3_)

**Fig. 16S** CD spectrum of compound **2**

**Fig.17S** MS spectrum of compound **2**

**Fig. 1S** ^1^H NMR spectrum of compound **1** (DMSO-*d_6_*, 500 MHz)
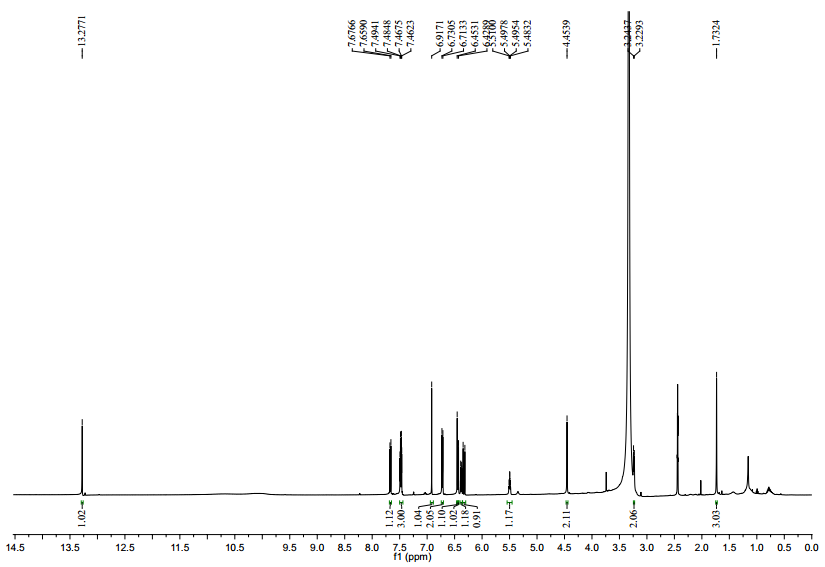


**Fig. 2S** ^13^C NMR spectrum of compound **1** (DMSO-*d_6_*, 125 MHz)
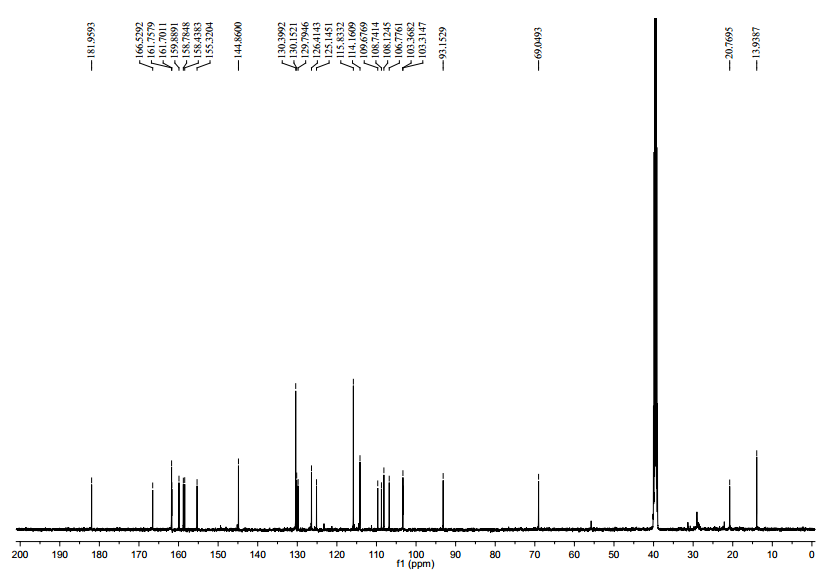


**Fig. 3S** DEPT spectrum of compound **1** (DMSO-*d_6_*)
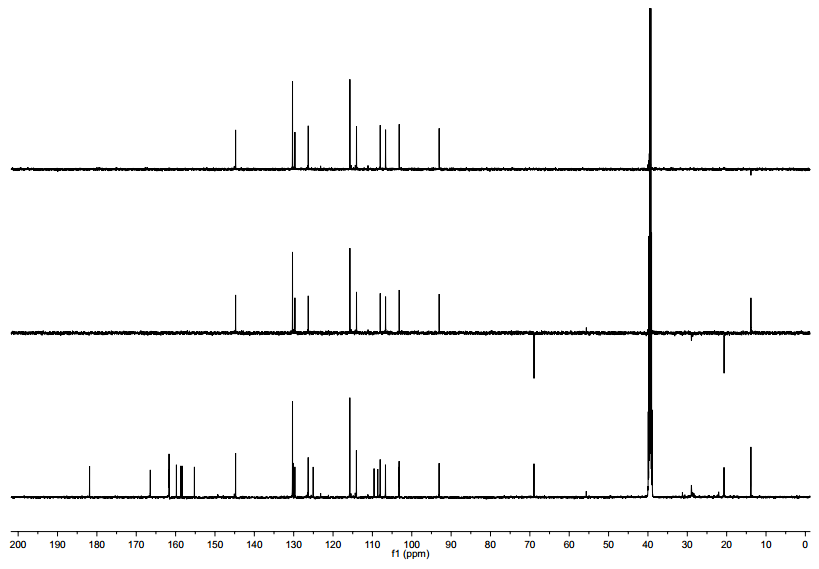


**Fig. 4S** COSY spectrum of compound **1** (DMSO-*d_6_*)
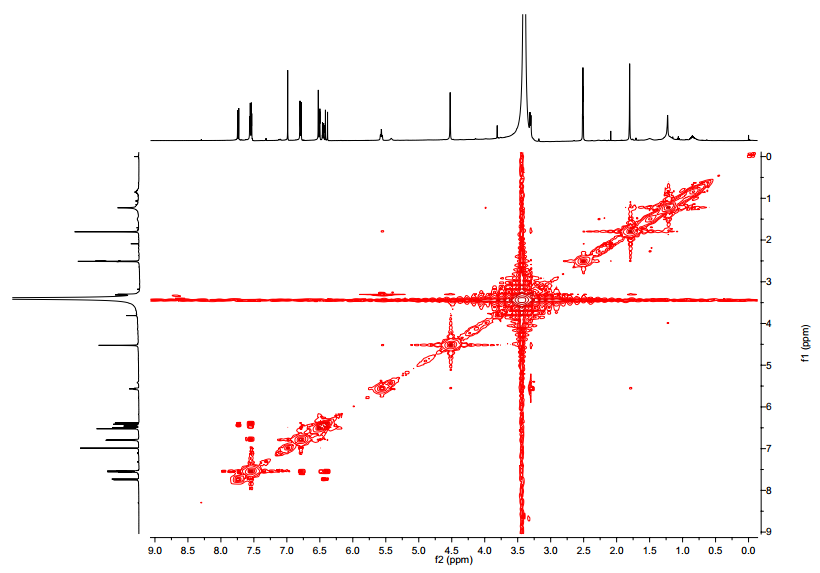


**Fig. 5S** HSQC spectrum of compound **1** (DMSO-*d_6_*)
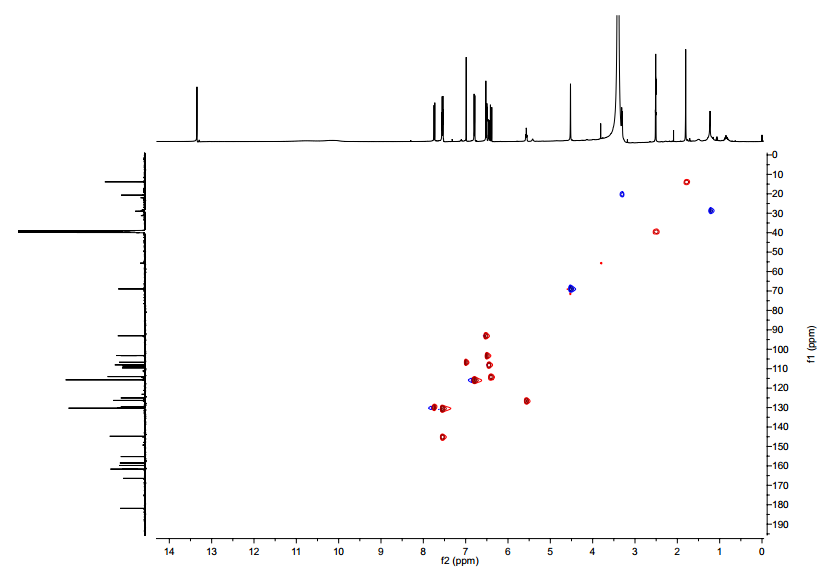


**Fig. 6S** HMBC spectrum of compound **1** (DMSO-*d_6_*)
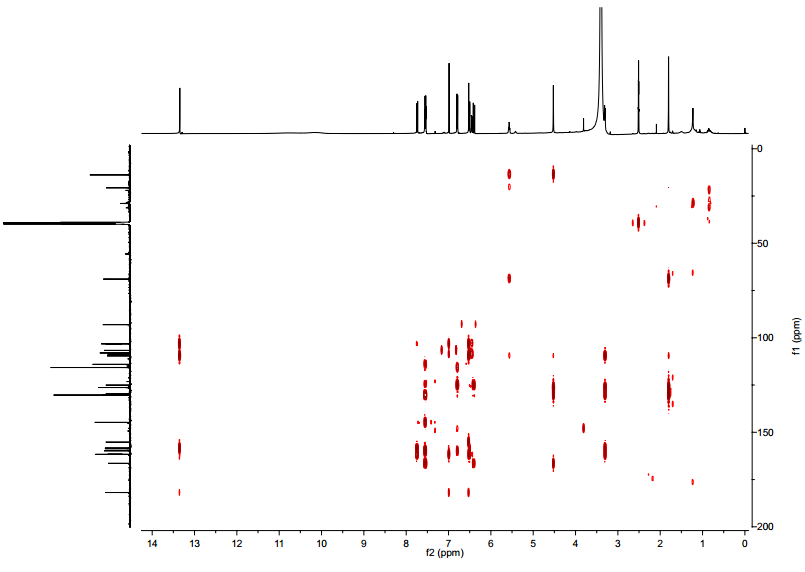


**Fig. 7S** NOESY spectrum of compound **1** (DMSO-*d_6_*)
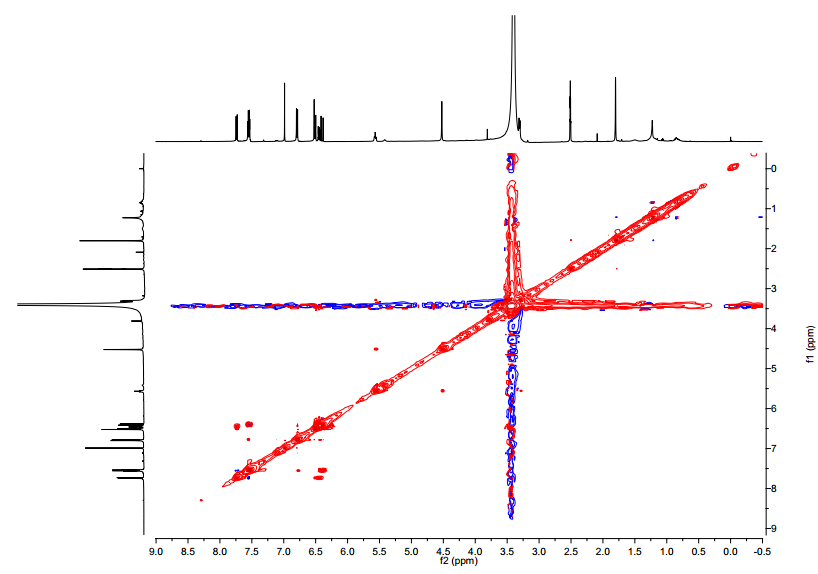


**Fig.8S** MS spectrum of compound **1**
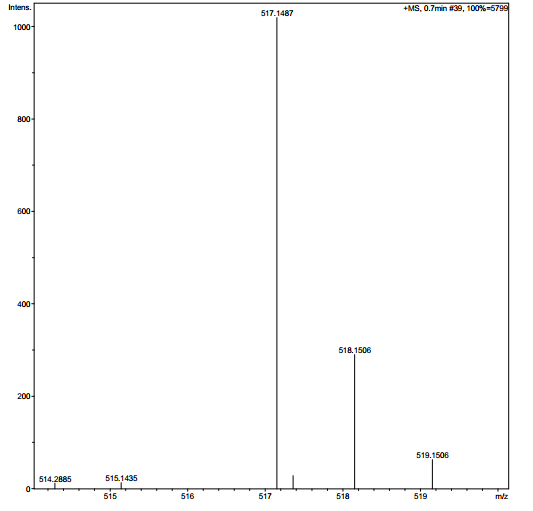


**Fig. 9S** ^1^H NMR spectrum of compound **2** (CD_3_COCD_3_, 500 MHz)
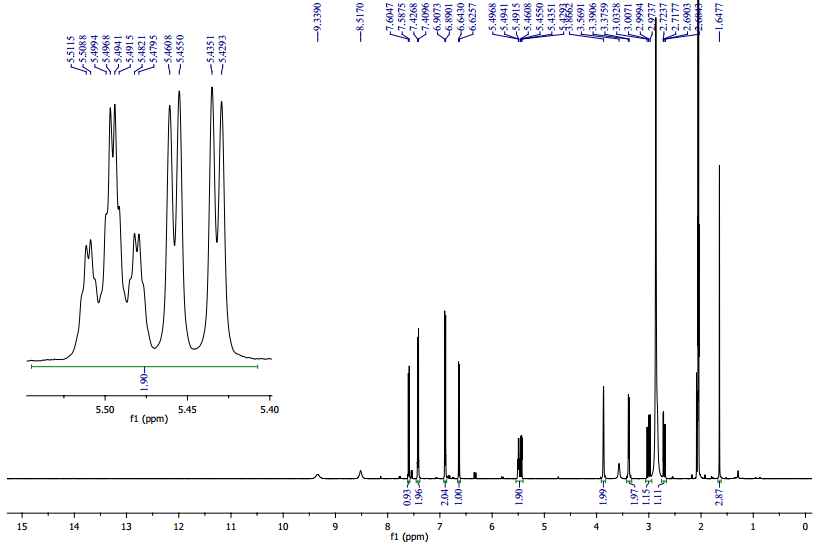


**Fig. 10S** ^13^C NMR spectrum of compound **2** (CD_3_COCD_3_, 125 MHz)
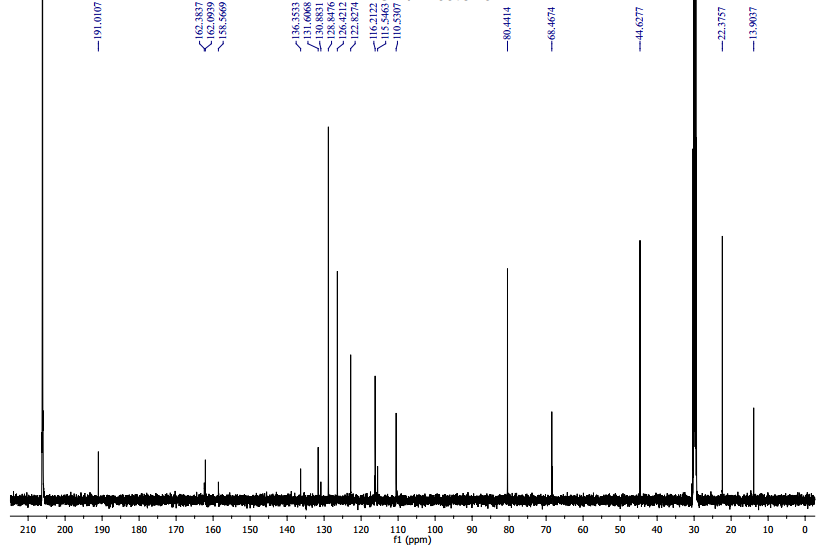


**Fig. 11S** DEPT spectrum of compound **2** (CD_3_COCD_3_)
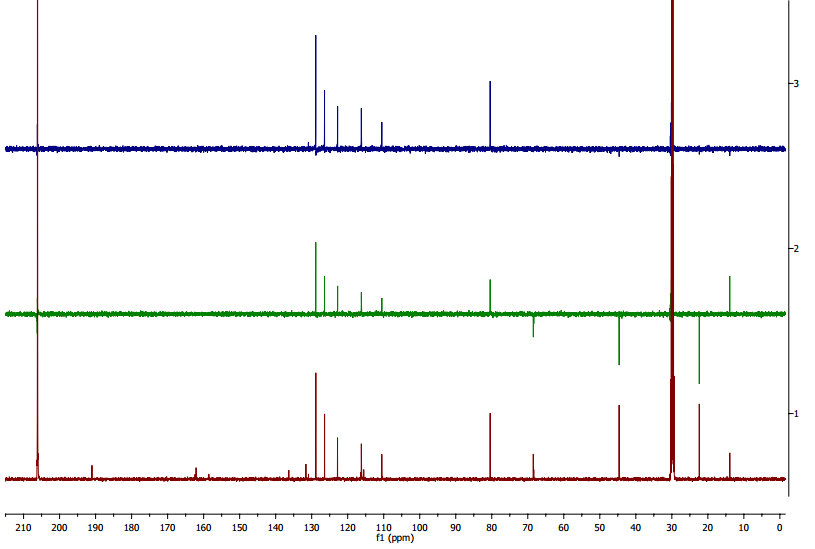


**Fig. 12S** COSY spectrum of compound **2** (CD_3_COCD_3_)
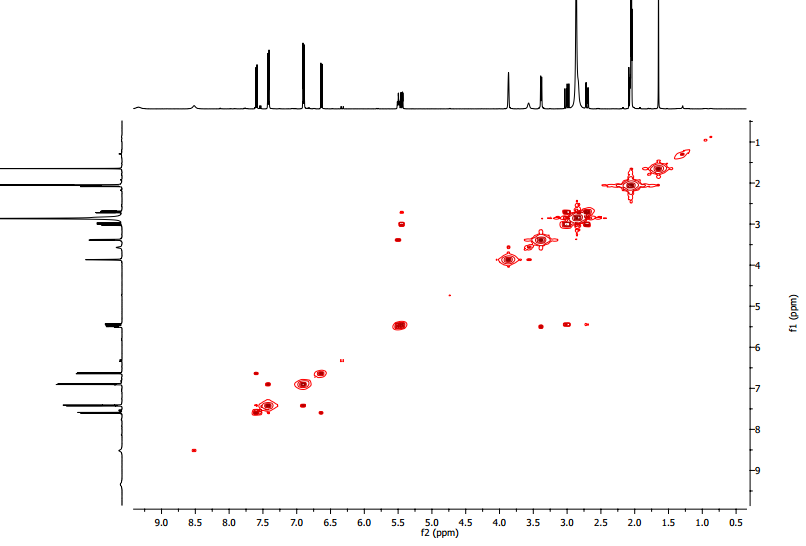


**Fig. 13S** HSQC spectrum of compound **2** (CD_3_COCD_3_)
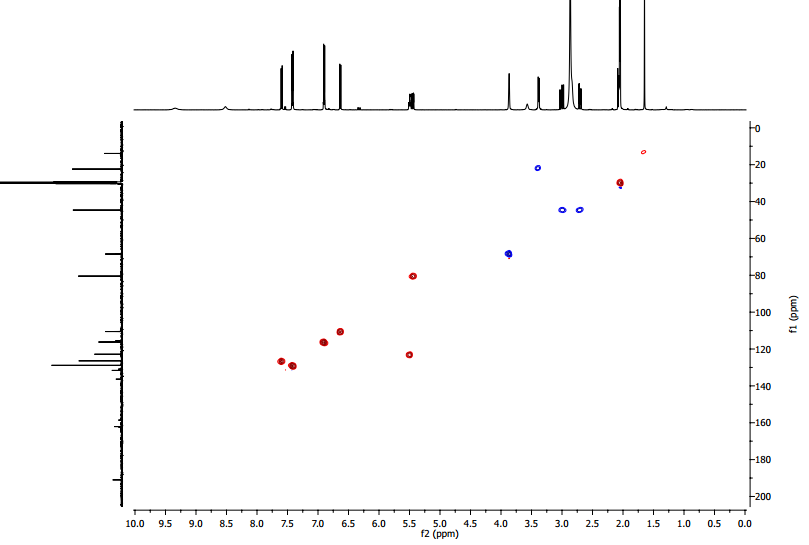


**Fig. 14S** HMBC spectrum of compound **2** (CD_3_COCD_3_)
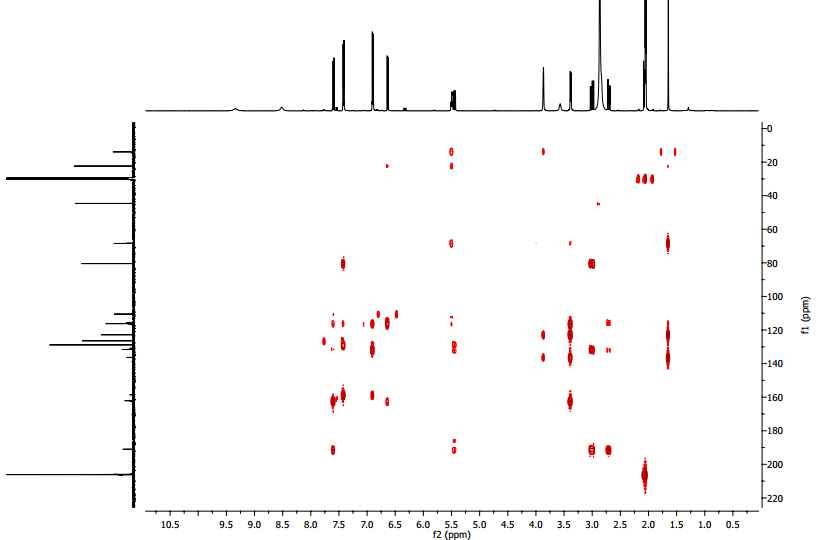


**Fig. 15S** NOESY spectrum of compound **2** (CD_3_COCD_3_)
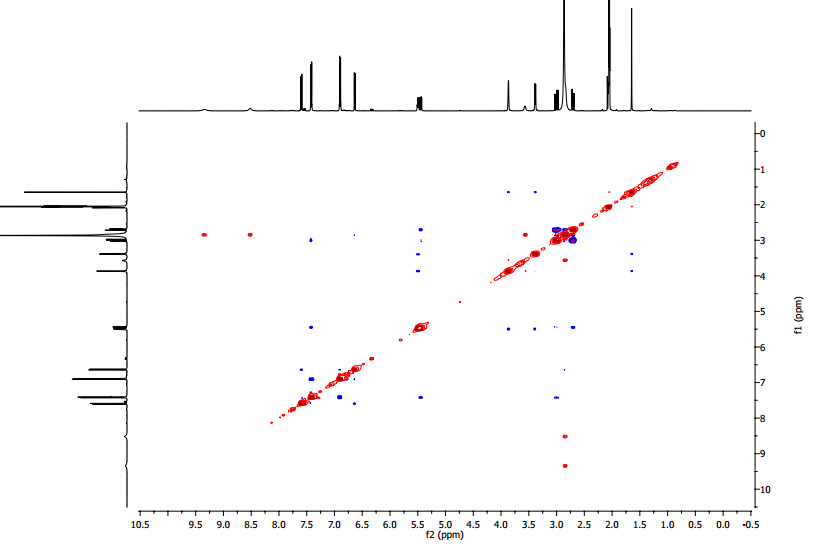


**Fig. 16S** CD spectrum of compound **2**


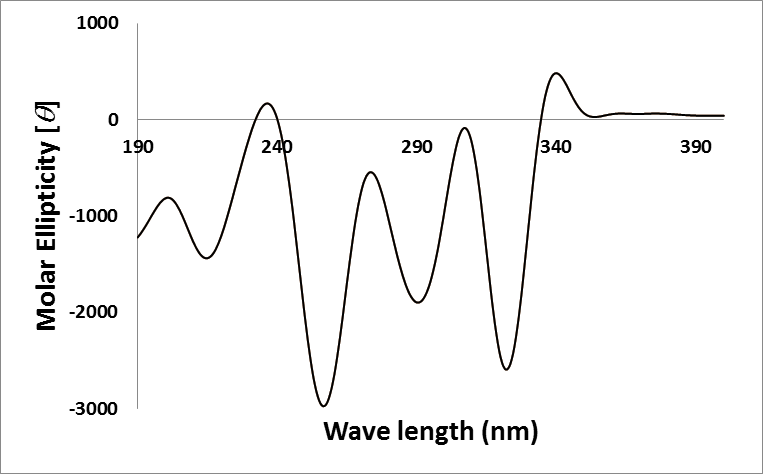


**Fig.17S** MS spectrum of compound **2**
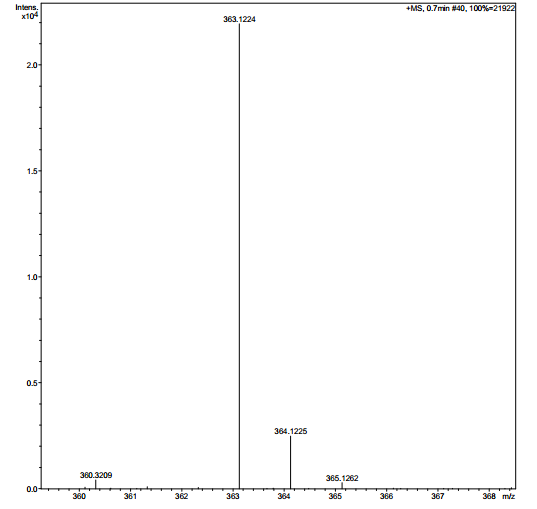

Supplement: Supplementary file 1 — 10.1186/s13065-016-0150-7 One-dimensional (1D) and two-dimensional (2D) nuclear magnetic resonance (NMR) andmass spectrometry (MS) of compounds 1-2 [file 13065_2016_150_MOESM1_ESM.doc]
